# Childhood symptoms of attention‐deficit/hyperactivity disorder and borderline personality disorder

**DOI:** 10.1111/acps.13476

**Published:** 2022-07-23

**Authors:** Annika Tiger, Anna Ohlis, Johan Bjureberg, Sebastian Lundström, Paul Lichtenstein, Henrik Larsson, Clara Hellner, Ralf Kuja‐Halkola, Nitya Jayaram‐Lindström

**Affiliations:** ^1^ Centre for Psychiatry Research, Department of Clinical Neuroscience Karolinska Institutet & Stockholm Health Care Services, Region Stockholm Stockholm Sweden; ^2^ Centre for Epidemiology and Community Medicine & Stockholm Health Care Services Region Stockholm Stockholm Sweden; ^3^ Department of Psychology Stanford University Stanford California USA; ^4^ Department of Neuroscience and Physiology Sahlgrenska Academy Göteborg Sweden; ^5^ Department of Medical Epidemiology and Biostatistics Karolinska Institutet Stockholm Sweden; ^6^ Department of Medical Sciences Örebro University Örebro Sweden

**Keywords:** ADHD, borderline personality disorder, impulsivity, longitudinal studies, symptomatology

## Abstract

**Objective:**

Childhood attention‐deficit /hyperactivity disorder (ADHD) is known to be associated with adult Borderline Personality Disorder (BPD). We investigated if any of the subdimensions of childhood ADHD, that is, impulsivity, inattention, or hyperactivity was more prominent in this association.

**Methods:**

In a nation‐wide cohort (*N* = 13,330), we utilized parent reported symptoms of childhood ADHD and clinically ascertained adult BPD diagnoses. The summed total scores of ADHD symptoms and its three subdimensions were used and standardized for effect size comparison. Associations were analyzed using Cox regression with sex and birth‐year adjustments. Secondary outcomes were BPD‐associated traits (i.e., self‐harm and substance use) analyzed using logistic‐ and linear regression respectively.

**Results:**

ADHD symptom severity was positively associated with BPD with a hazard ratio (HR) of 1.47 (95% confidence interval [CI]: 1.22–1.79) per standard deviation increase in total ADHD symptoms. Impulsivity was the most prominent subdimension with the only statistically significant association when analyzed in a model mutually adjusted for all ADHD subdimensions—HR for inattention: 1.15 (95% CI: 0.85–1.55), hyperactivity: 0.94 (95% CI: 0.69–1.26), impulsivity: 1.46 (95% CI: 1.12–1.91). In secondary analyses, weak positive associations were seen between total ADHD symptom score and self‐harm and substance use. In analyses by subdimensions of ADHD, associations were weak and most prominent for inattention in the model with self‐harm.

**Conclusion:**

Childhood ADHD symptoms were associated with subsequent development of BPD diagnosis and appeared to be driven primarily by impulsivity. Our findings are important for understanding the association between childhood symptoms of ADHD and subsequent BPD.

**Significant Outcomes**

- Results indicate that impulsivity impacts the association between childhood symptoms of ADHD and BPD the most out of the subdimensions of ADHD, that is, inattention, hyperactivity and impulsivity.
- Clinical implications include the importance in noting individual symptom profiles in children. More specifically it highlights the importance in noting children with impulse control difficulties regardless of ADHD‐diagnosis status with regards to BPD, particularly in girls with other predisposing factors for BPD.
- In practice, our finding could be helpful for clinicians and policymakers to make informed decisions when prioritizing health care resources, for example, to prioritize girls with impulse control difficulties for follow up.

**Limitations**

- Individuals diagnosed with BPD are predominantly female in our sample, as previously found in clinical populations, whereas more evenly prevalent in the two sexes in population studies. This may limit the generalizability of our findings to male.
- We were unable to analyze the associations between childhood symptoms of ADHD and later BPD in a subsample of individuals with diagnosed ADHD because of limitation by power. This may limit generalizability to a clinical population of individuals with diagnosed ADHD.

## 1 INTRODUCTION

Borderline personality disorder (BPD) is a condition characterized by instability in affect, self‐image, and interpersonal relationships and by extreme sensitivity to perceived interpersonal slights.^1^, ^2^, ^3^ Self‐destructive behaviors, such as self‐harm and substance use are common and often further aggravate the underlying condition.^2^, ^3^, ^4^, ^5^ The symptoms and difficulties of BPD are particularly prevalent during adolescence and early adulthood.^6^ BPD and its associated psychiatric and social consequences are associated with significant suffering and studies have reported lifetime BPD prevalence estimated to approximately 6%.^7^ Health care consumption is disproportionally high alongside high rates of suicide (4.7%^8^) in this group of patients.

Attention‐deficit/hyperactivity disorder (ADHD) is a neuropsychiatric condition with childhood‐onset characterized by impairment because of difficulties with sustaining attention, hyperactivity, and impulse control.^3^ ADHD is a common comorbid condition in individuals with BPD^9^, ^10^, ^11^ and the two conditions share symptoms of impulsivity and emotional instability.

Etiological studies have shown genetic and environmental causes for BPD.^9^, ^12^ There are also indications in the literature of ADHD and BPD sharing origin in terms of genetic and environmental factors.^13^, ^14^ It has been suggested that ADHD could be an early stage of BPD^15^ and that the two conditions constitute different manifestations of a common underlying mechanism.^16^ This is supported by findings of children diagnosed with ADHD being at increased risk of developing BPD^17^, ^18^ and of ADHD‐symptom severity in childhood being positively associated with BPD‐symptom severity in adulthood.^19^, ^20^ However, little is known if any subdimension of childhood ADHD is particularly prominent in the association with later BPD.

Both the international classification of disorders (ICD)^21^, ^22^and the Diagnostic and Statistical Manual of Mental Disorders (DSM)^3^ acknowledge three symptom dimensions in ADHD; the inattentive, the hyperactive and the impulsive, and previous studies of how the symptoms of ADHD covary have supported this three‐factor structure.^23^, ^24^ However, most research to date has focused on ADHD subdivided into either the inattentive subdimension or the hyperactive and impulsive subdimension or all combined as defined in the DSM.^25^, ^26^

To our knowledge only one previous study has attempted to investigate if the subdimensions of ADHD during childhood are differentially present in individuals with BPD.^27^ That study aimed to identify classes of different profiles of childhood symptoms of ADHD and accompanying adult symptoms of BPD and/or ADHD. The findings suggested ten different developmental pathways between specific childhood ADHD symptom combinations and adult BPD‐ and ADHD symptom combinations. The most common pathway was between childhood symptoms of inattention, hyperactivity as well as impulsivity and subsequent adult symptoms of inattention, hyperactivity and impulsivity plus high levels of symptoms of BPD. However, symptoms of ADHD in childhood were assessed in retrospect in this study, inducing risk of recall bias. In addition, the population was a clinical sample of adults diagnosed with BPD and/or ADHD without general population controls. The finding of developmental pathways between specific childhood ADHD symptom profiles and subsequent adult BPD/ADHD symptom profiles implied a value in further investigating the association between the subdimensions of ADHD and BPD.

### 1.1 Aims of the study

The primary aim was to investigate if any of the subdimensions of ADHD in childhood (i.e., inattention, hyperactivity or impulsivity) was more prominent in the association between childhood symptoms of ADHD and clinically ascertained BPD. The secondary aim was to investigate if any of those subdimensions was more prominently associated with BPD‐related behaviors, specifically self‐harm, alcohol use, and drug use. Our hypothesis was that impulsivity would consistently be most prominently associated with all outcome measures.

## 2 METHODS

### 2.1 Study population

We used data from the Child and Adolescent Twin Study in Sweden (CATSS), which invites parents of all twins born in Sweden from July 1, 1992 and onwards.^28^ We included twins whose parents had responded to the assessment of ADHD‐symptoms in their children at age 9 or 12 (individuals in the first three birth cohort years were contacted at age 12, the rest at age 9). The overall participation rate in the age 9/12 assessment was 69% (75% in the first birth cohorts).^29^ We also used assessments at age 18 and national register data, linked through the individually unique Swedish personal numbers. See Figure S1 for study cohort selection.

Information about BPD diagnoses were extracted from the National Patient Register (NPR)^30^ updated until December 31, 2016. CATSS data were updated until August 29, 2019.

In the primary outcome analyses investigating associations between childhood symptoms of ADHD and adult BPD, individuals younger than 18 at the last NPR update were excluded as BPD is generally not diagnosed before age 18 in Swedish clinical practice (see e.g. Supplemental eFigure 1 in Skoglund et al.^9^).

In secondary analyses the outcome measures were self‐harm and quantity of alcohol‐ and drug use utilizing data from the CATSS self‐assessments at age 18. Individuals who had not responded to all the age 18 assessments utilized in our study were excluded in these analyses.

The study cohort consisted of 13,330 individuals in the primary analyses and 8995 individuals in secondary analyses (see Figure S1 for information on inclusions and exclusions).

### 2.2 Exposure variables

At age 9 or 12, parent rated childhood ADHD symptoms were assessed using the Autism—Tics, AD/HD and other Comorbidities inventory (A‐TAC), evaluating the degree of the 18 symptoms of ADHD as defined in DSM IV^31^, ^32^ and ICD 10.^2^, ^33^ The A‐TAC‐items have three response options 0 = No, 0.5 = Yes, to a certain degree, and 1 = Yes, refer to lifetime symptoms and have been found to be of good reliability^34^ and of good validity^31^ regarding clinically diagnosed ADHD. We constructed a sum score of *total ADHD‐symptoms*, and three of the subdimensions of ADHD—*inattention* (9 questions), *hyperactivity* (5 questions) and *impulsivity* (4 questions), according to the ICD‐10 definition^22^, ^33^—a subdivision previously confirmed through factor analysis.^31^ We confirmed the same tree‐factor structure, using an exploratory approach with the principal component method, with an additional approximately 15,000 individuals compared with the original report^31^ (see Figure S2 and Table S1 for results from the exploratory factor analysis with the principal component method). The ADHD‐scores and subdimensions were standardized before analyses to facilitate effect size comparison.

### 2.3 Outcome variables

We obtained the first recorded date of diagnosed *BPD*, defined as an F60.3 ICD‐10‐code in the NPR. We did not differentiate between primary‐ and secondary diagnoses. The diagnoses registered in the NPR are based on clinical assessments. The national clinical guidelines for assessment and treatment of personality disorder issued by the Swedish Psychiatric Association recommends careful clinical interviews, using validated interview instruments for assessing BPD and SCID‐II is emphasized.^35^ Two medical chart validation studies have been performed regarding BPD diagnosis in the NPR; a study by Kuoppis et al. found a positive predictive value of 100% using ICD‐criteria,^36^ another study by Kuja‐Halkola et al. reported a positive predictive value of 81%.^14^

We created a binary *self‐harm* variable defined as having or not having self‐harmed up to the age of 18 based on the combination of self‐ratings in the Life History of Aggression questionnaire (LHA), and the Brief Obsessive Compulsive Scale (BOCS), and parental ratings in the Adult Behavior Check List (ABCL). See Table S2 for questionnaire details and Table S3 for distribution of responses.

Sum scores of *alcohol* and *drug use*, were created from self‐ratings in the Alcohol Use Disorders Identification Test (AUDIT) and the Drug Use Disorders Identification Test (DUDIT), respectively (see Table S2).

### 2.4 Covariates

*Sex* was considered a confounder and *birthyear* was included to take diagnostic trends into account. Medication for ADHD before the childhood assessment was considered a possible confounder reducing both childhood ADHD symptoms and adult BPD symptoms. ADHD‐medication before BPD or secondary outcomes were considered possible mediators as we expected the degree of ADHD‐symptoms in childhood to be positively associated with medication status, which in turn was expected to be associated with reduced symptoms of BPD compared with individuals with similar childhood ADHD‐symptoms who remained un‐medicated. Therefore, binary variables, indicating having or not having medicated with drugs for ADHD, were created with data from the Swedish Prescribed Drug Register.^37^ See Table S3 for detailed information.

ADHD‐symptoms at age 18 were self‐rated in the ADHD Self‐Report Scale (ASRS)^38^ and were considered possible mediators. Sum scores equivalent to those based on childhood assessment were therefore created (Table S2).

Ethical approval was obtained from the Swedish Ethical Review Authority, Stockholm (Dnr: 2016 2135–31 and 2020–04962). All participants have given informed consent.

### 2.5 Statistical analyses

#### 2.5.1 Descriptive information

We summarized the total number and percentages of individuals with BPD diagnosis, means and numbers and proportions of the exposure and outcome variables, the numbers and proportions of those who had had medication in childhood and medication before the outcomes, and the means of the ADHD‐symptomatology at age 18. We did this for the total population and by sex. We also estimated the cumulative incidence of BPD, for the total population and stratified by sex, using Kaplan–Meier approach.

#### 2.5.2 Association with BPD diagnosis

To analyze the association between childhood ADHD‐symptoms and subsequent BPD diagnoses we used Cox regression and estimated hazard ratios (HR) with 95% confidence intervals (CIs). We performed separate analyses for the total ADHD‐symptoms and for each subdimension (inattention, hyperactivity, and impulsivity) to analyze the effect of each exposure. To compare the effects of each subdimension to each other, we also fitted a mutually adjusted model. The estimates were adjusted for sex and birth year. Since most individuals with BPD diagnoses in our sample were female and the NPR BPD diagnoses in females have been found to be of good validity, we additionally performed the same analyses among females only.

#### 2.5.3 Association with secondary outcomes

To analyze associations between childhood ADHD symptoms and secondary BPD‐associated outcomes up until age 18, we used logistic regression to estimate odds ratios (ORs) of self‐harm, and linear regression to estimate associations with alcohol‐ and drug use. Analogous to analyses with BPD as outcome, separate, and mutually adjusted models were fitted. All analyses were adjusted for sex and birth year.

#### 2.5.4 Sensitivity analyses

For primary outcome analyses of BPD, analyses with adjustment for medication before BPD and adult ADHD symptoms were performed. For secondary outcomes additional analyses with adjustments for medication in childhood and before outcomes as well as for adult ADHD symptoms were performed.

To account for shared, stable confounders (i.e., socioeconomic background and psychiatric heredity) we performed within‐pair analyses, where twins were compared with their co‐twins,^9^, ^39^ separately by zygosity. We used stratified Cox regression in analyses of BPD, conditional logistic regression in analyses of self‐harm and conditional linear regression in analyses of alcohol‐ and substance use.

Cluster robust sandwich estimator (for standard errors) was used in all analyses to account for deviations from modeling assumptions and dependence between observations in twin pairs. All analyses were performed in R using the “survival”^40^ and “dergee”^41^ packages. A *p*‐value < 0.05 was considered statistically significant.

## 3 RESULTS

### 3.1 Descriptive

Table 1 shows the characteristics of our study population (*N* = 13,330). The overall sex distribution was even [female: 48.7% (*N* = 6489), male: 51.3% (*N* = 6841)]. Childhood mean ADHD‐symptom scores were higher in males than in females. Individuals with BPD diagnoses were predominantly female (46 out of 49 individuals). Of the entire population, 29.1% (2622 individuals) had self‐harmed up until age 18, which was more common among females (35.1%) than males (21.0%). AUDIT and DUDIT mean scores indicated higher alcohol and drug consumption among males than females.

**TABLE 1:** Distribution of variables in the study population

		Total population	Female	Male
Full cohort, *N*		13,330	6489	6841
Exposure variables at age 9/12	ADHD total score, mean (SD)	1.8 (2.9)	1.3 (2.4)	2.2 (3.2)
	Inattention, mean (SD)	1.0 (1.7)	0.8 (1.5)	1.3 (1.9)
	Hyperactivity, mean (SD)	0.3 (0.8)	0.2 (0.6)	0.4 (0.9)
	Impulsivity, mean (SD)	0.5 (0.8)	0.4 (0.8)	0.5 (0.9)
Outcome variables	BPD‐diagnosis, *n* (%)	49 (0.4)	46 (0.7)	3 (0.0)
	Self‐harm, *n* (%)^a^	2622 (29.1)	1827 (35.1)	795 (21.0)
	AUDIT, mean (SD)^a^	4.7 (4.2)	4.5 (4.0)	5.0 (4.5)
	DUDIT, mean (SD)^a^	0.3 (1.7)	0.2 (1.6)	0.4 (1.9)
Medication	Medication before examination at age 9/12, *n* (%)	40 (0.3)	8 (0.1)	32 (0.5)
	Medication ever, *n* (%)	483 (3.6)	140 (2.2)	343 (5.0)
Birth cohorts	1992, *n* (%)	1022 (7.7)	487 (7.5)	535 (7.8)
	1993, *n* (%)	2285 (17.1)	1118 (17.2)	1167 (17.1)
	1994, *n* (%)	2121 (15.9)	1055 (16.3)	1066 (15.6)
	1995, *n* (%)	2063 (15.5)	971 (15.0)	1092 (16.0)
	1996, *n* (%)	1938 (14.5)	943 (14.5)	995 (14.5)
	1997, *n* (%)	2047 (15.4)	974 (15.0)	1073 (15.7)
	1998, *n* (%)	1854 (13.9)	941 (14.5)	913 (13.3)
Zygosity	Monozygotic, *n* (%)	3968 (29.8)	2058 (31.7)	1910 (27.9)
	Dizygotic same sex, *n* (%)	4772 (35.8)	2150 (33.1)	2622 (38.3)
	Unknown zygosity, *n* (%)	143 (1.1)	57 (0.9)	86 (1.3)
	Dizygotic opposite sex, *n* (%)	3334 (34.3)	2223 (32.5)	4447 (33.4)

*Note*: Cohort subjects followed from age 9/12 examination until the last update in the National Patient Register‐data (December 31, 2016). Birth cohorts: 1992–1998, *N* = 13330.

^a^ Cohort subjects examined at age 9/12 for ADHD‐symptomatology and at age 18 for self‐harm, alcohol‐ and drug use. Birth cohorts: 1992–2000, *n* = 8995.

**FIGURE 1:**
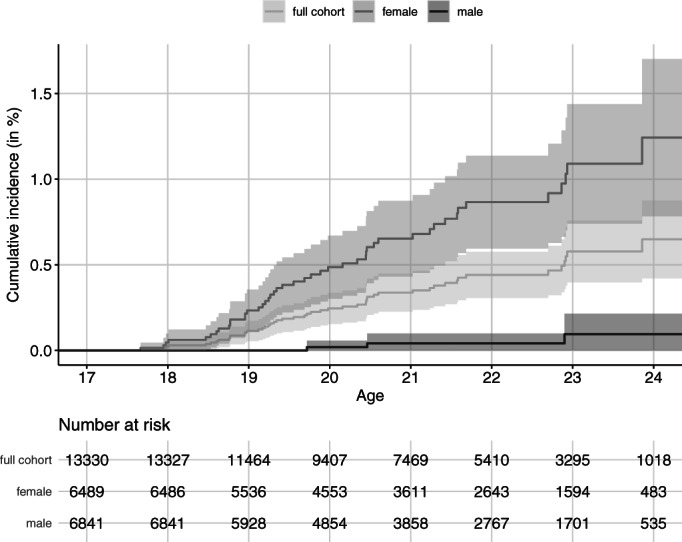
Cumulative incidence of BPD in full cohort and in and in female and male separated. Kaplan–Meier estimates with 95% confidence intervals

Figure 1 shows the cumulative incidence of BPD that was approximately 0.7% (1.3% in female and 0.1% in male) at age 24.5.

### 3.2 Association with BPD diagnosis

**FIGURE 2:**
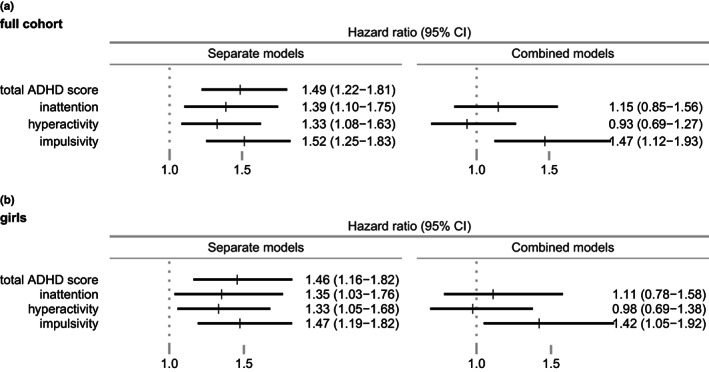
Hazard ratios and 95% CI of BPD by standard deviation increase in ADHD symptoms. Separate models on left side. Combined, mutually adjusted models on right side

Childhood ADHD symptom severity was positively associated with later BPD, see Figure 2 for estimates and 95% CIs. The HR by standard deviation increase in total ADHD score was 1.49 (95% CI: 1.22–1.81). When analyzing by subdimensions, they were each separately positively associated with BPD, HRs 1.39 (95% CI: 1.10–1.75) for inattention, 1.33 (95% CI: 1.08–1.63) for hyperactivity and 1.52 (95% CI: 1.25–1.83) for impulsivity, per standard deviation increase in symptom score. When the subdimensions were analyzed in a combined, mutually adjusted model, inattention (HR: 1.15 [95% CI: 0.85–1.56]) and hyperactivity (HR: 0.93 [95% CI: 0.69–1.27]) were weaker and not statistically significant, while the strength of the association with impulsivity remained (HR: 1.47 [95% CI: 1.12–1.93]).

We observed the same pattern in sub‐group analyses of BPD in females (Figure 2). Analyses in male only were omitted because of insufficient data.

### 3.3 Association with secondary outcomes

**FIGURE 3:**
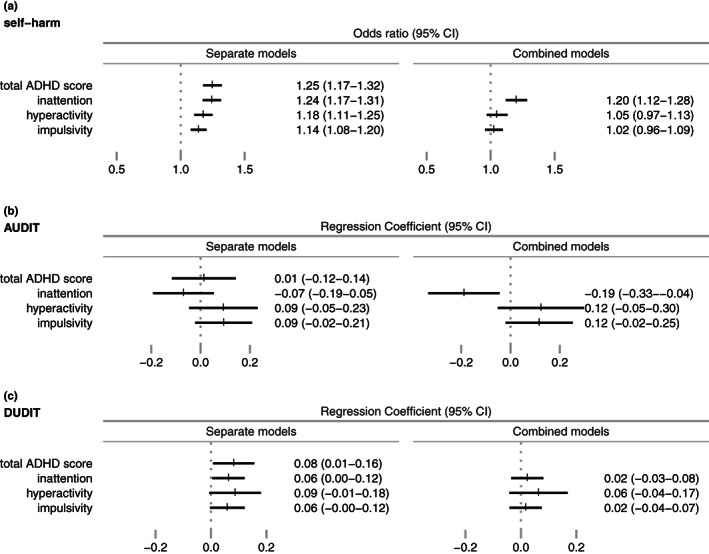
Odds ratio and Regression Coefficients with 95% CI by standard deviation increase in ADHD symptoms. Separate models on left side. Combined, mutually adjusted models on right side

For the BPD‐associated behaviors we found positive associations between total ADHD‐score and self‐harm, OR: 1.25 (95% CI: 1.17–1.32) (Figure 3). When examining the associations by subdimension in separate models, the most prominent positive association was with inattention (OR: 1.24 [95% CI: 1.17–1.31]) and the tendency remained in the combined model (OR: 1.20 [95% CI: 1.12–1.28]).

The results indicated no statistically significant association between total ADHD symptom score and alcohol use, as measured with AUDIT, and a negative association between inattention and alcohol use when the subdimensions were combined in one mutually adjusted model. A weak positive association with total ADHD‐score and drug use, as measured with DUDIT, was found (regression coefficient: 0.08 [95% CI: 0.01–0.16], i.e., 0.08 more drug use symptoms endorsed per standard deviation increase in total ADHD‐score), but no statistically significant positive association by any of the subdimensions.

### 3.4 Sensitivity analyses

Sensitivity analyses with additional adjustment for medication before BPD and ADHD‐symptoms at age 18 did not alter the overall tendencies in models with BPD. The patterns remained also in sensitivity analyses with adjustment for medication for ADHD in childhood and before outcomes and for age 18 symptoms in models with self‐harm, AUDIT and DUDIT. In within twin‐pair analyses with BPD and secondary outcomes, all associations were attenuated with wide confidence intervals and no statistically significant findings. See Figure S3 for estimates with 95% CIs in sensitivity analyses.

## 4 DISCUSSION

To our knowledge this is the first longitudinal study investigating the association between the childhood ADHD subdimensions of inattention, hyperactivity, and impulsivity and later BPD. The results of the present study confirm results from previous studies of a positive association between the degree of total ADHD‐symptom severity in childhood and later BPD. The subdimension analyses indicate that childhood symptom of impulsivity is the most prominent of the subdimensions, and drives the association with later BPD.

Results from the secondary analyses indicate similar positive associations between total ADHD symptom score and self‐harm and drug use but not with alcohol use. Unlike the overall association with BPD, the most prominent subdimensional association was between inattention and self‐harm, but no positive association was found for any of the subdimensions with alcohol‐ or drug use. Lastly a weak negative association was found between inattention and alcohol use in the combined model.

In sensitivity analyses with adjustments for medication before BPD and for symptoms of ADHD at age 18, the overall association patterns persisted, indicating that these factors do not influence the associations. Within‐pair analyses indicated that genetic and environmental effects shared between twins may play a role in the associations however, caution is warranted because of limited statistical power.

The results of the current study confirm previously raised hypotheses of impulsivity as a common underlying feature in ADHD and BPD.^42^ This is in line with neuroimaging data indicating aberrant prefrontal function associated with impaired impulse control in both BPD and ADHD in childhood as well as in adulthood.^43^, ^44^

Clinical implications include the importance of noting individual symptom profiles. Specifically, our findings indicate a value in noting the subdimensions of ADHD as separate entities as associations differ between those and BPD and the associated behaviors self‐harm, alcohol‐ and drug use. For instance, for BPD the results indicate that impulsivity is the most prominent and driving of the subdimensions in the association with childhood symptoms of ADHD, whereas for self‐harm, inattention is the most prominent and driving of the subdimensions.

Regarding self‐harm, the result deviates from the hypothesis and previous findings^25^, ^45^ where childhood hyperactive/impulsive ADHD subtype has been found to be associated with higher HR of suicide attempts. In contrast to previous studies based on the two subdimensions inattention and hyperactivity/impulsivity, we analyzed hyperactivity and impulsivity as a separate subdimensions which may explain the differences in findings. The manner in which the self‐harm variable was constructed may also be of importance to the current finding. The rationale in using CATSS‐questionnaire data was to use a sensitive measure of self‐harm and the assumption that self‐rating measures are more sensitive than, for example, clinical diagnoses. For instance, not all individuals who self‐harm seek health care and self‐rating measures are likely to capture such behaviors at lower thresholds than routine clinical interviews.^46^ Also, in contrast to merely studying association with suicide attempts, we analyzed associations with a combination of non‐suicidal self‐injury (NSSI) and suicide attempts. This decision was made because suicidal intent is often transient and unreliable and as both behaviors are part of the diagnostic criteria for BPD and constitute important risk factors for death by suicide.^47^ Our prevalence estimates of self‐harm are in‐line with previous Swedish population estimates,^46^ indicating validity and thereby generalizability of the present findings. In a clinical context these findings indicate that it may be of particular importance to assess self‐harm in individuals with problems of inattention. The understanding of the association between subdimensions of ADHD and self‐harm may benefit from further studies of NSSI and suicide attempts separately.

Of specific interest, the only statistically significant associations regarding childhood ADHD symptoms and quantity of alcohol‐ and drug use was a weak positive association between total ADHD‐score and DUDIT score and a negative association between inattention and AUDIT score. These findings are surprising, as they differ from previous studies where total ADHD score and the hyperactive/impulsive subtype has been found to be significantly associated with AUDIT^26^ and ADHD and impulsivity has been found to be associated with alcohol‐ and drug use.^48^ Possible explanations for this difference, may lie in the variable construction and study design. The decision to use self‐rated AUDIT and DUDIT data from CATSS for these two secondary outcomes was based on the same rationale as for the self‐harm variable, that is, aiming for sensitive measures of substance use. The clinical scales AUDIT and DUDIT were designed for early detection of harmful use.^49^ The study design only captures problematic alcohol‐ and drug use around the age of 18 and not with later onset. Also, individuals with severe mental illness including alcohol‐ and drug use disorders are under‐represented in the CATSS cohort.^28^ In contrast to previous studies, we analyzed the hyperactive and impulsive subdimensions separately and not together. Taken together, the estimates for alcohol‐ and substance use should be interpreted with caution.

There are several strengths to our study. The study population is large (*N* = 13,330 in analyses of BPD and 8995 in secondary analyses) and nation‐wide, enabling outcome estimates of good precision and generalizability. The instrument used for measuring ADHD symptoms, A‐TAC, has a good reliability and validity adding to precision and accuracy respectively. BPD‐diagnoses in the NPR have good validity adding to accuracy. Also, the longitudinal design with prospective assessments reduces recall bias.

Some limitations need to be mentioned. Regarding our study population: A family history of psychiatric illness, lower socioeconomic status and diagnosed ADHD is more common among nonparticipants.^28^ Individuals diagnosed with BPD are predominantly female in our sample, as previously found in clinical populations and in the NPR,^9^, ^36^ whereas more evenly prevalent in the two sexes in studies based on face‐to‐face interviews in samples representative of general populations.^7^ The cumulative incidence of BPD of 0.7% in the current study is low, compared with that of general population interview studies,^7^ particularly in men (0.1%) and those oldest at study end are 24.5 years old. Even so, the estimates are in line with previous clinical population estimates of a birth cohort that overlaps with our study population (see supplemental eFigure 1 in Skoglund et al.^9^). Taken together; the population, the selection and variable definition, may limit generalizability particularly in male and in individuals with BPD symptomatology with later onset. Regarding the ADHD symptom assessments at age 18 we lack information related to other factors such as comorbidity and substance use that may influence the reported symptoms, yet the association patterns persisted after adjustment for ADHD symptoms at age 18 indicating no major influence by those. Residual confounding cannot be ruled out in the associations that were found. This includes higher order psychiatric factors such as neuropsychiatric symptomatology in general or overall general psychopathology that could explain the associations between childhood symptoms of ADHD and later BPD.^50^, ^51^ Nevertheless, expanding the understanding of the association between childhood symptoms of ADHD and later BPD is important for potentially understanding how to reduce suffering and impairments related to BPD.

In future studies, we suggest investigating if the associations between childhood symptoms and ADHD and particularly impulsivity and later BPD is more pronounced in individuals with clinically ascertained ADHD compared with those without to potentially understand if this group should also be prioritized for neuropsychiatric evaluation. Because of the limited number of individuals with BPD this was not possible in our study. Further studies are needed to elucidate if treatments specifically targeting impulsivity, could reduce risks of later impairments related to BPD. In children having problems sustaining attention, our results indicate that assessing self‐harm may be particularly important but the associations between childhood ADHD symptom subdimensions and self‐harm, alcohol‐ and drug use need to be further studied.

## AUTHOR CONTRIBUTIONS

Annika Tiger, Anna Ohlis, Johan Bjureberg, Henrik Larsson, Clara Hellner, Ralf Kuja‐Halkola and Nitya Jayaram‐Lindström were involved in the conception, development and designing of the work. Ralf Kuja‐Halkola and Annika Tiger performed the analyses and drafted the work. Annika Tiger, Anna Ohlis, Johan Bjureberg, Sebastian Lundström, Paul Lichtenstein, Henrik Larsson, Clara Hellner, Ralf Kuja‐Halkola and Nitya Jayaram‐Lindström interpreted the results and provided critical revision on the manuscript. All authors take responsibility for the integrity of the data, accuracy of the data analyses and approved the final version of the work.

## CONFLICT OF INTEREST

The authors declare that there is no conflict of interest that could be perceived as prejudicing the impartiality of the research reported. H Larsson has served as a speaker for Medice, Evolan Pharma and Shire/Takeda and has received research grants from Shire/Takeda; all outside the submitted work.

### PEER REVIEW

The peer review history for this article is available at https://publons.com/publon/10.1111/acps.13476.

## Supporting information


**Appendix S1** Supporting Information

## Data Availability

The data that support the findings of this study cannot be shared publicly because of the Swedish Secrecy Act. Data from the national Swedish register and twin register were used for this study and made available by ethical approval. The data used for this study include: Swedish Twin Registry, National Patient Register, and the Prescribed Drug Register. Researchers may apply for access through the Swedish Research Ethics Boards (etikprovningsmyndigheten.se) and from the primary data owners: Swedish Twin Registry, and the National Board of Health and Welfare, in accordance with Swedish law.

## References

[1] Gunderson JG , Herpertz SC , Skodol AE , Torgersen S , Zanarini MC . Borderline personality disorder. Nat Rev Dis Primers. 2018;4:18029. 29795363 10.1038/nrdp.2018.29

[2] World Health Organization . The ICD‐10 classification of mental and Behavioural disorders. *Diagnostic Criteria for Research* 1993:123‐127. https://apps.who.int/iris/handle/10665/37108

[3] American Psychiatric Association . Diagnostic and Statistical Manual of Mental Disorders [Elektronisk Resurs]: DSM‐5. American Psychiatric Association; 2013.

[4] Fernandez KC , Jazaieri H , Gross JJ . Emotion regulation: a Transdiagnostic perspective on a new RDoC domain. Cognit Ther Res. 2016;40(3):426‐440. 10.1007/s10608-016-9772-2 PMC4979607 27524846

[5] Carpenter RW , Trull TJ . Components of emotion dysregulation in borderline personality disorder: a review. Curr Psychiatry Rep. 2013;15(1):335. 23250816 10.1007/s11920-012-0335-2 PMC3973423

[6] Bornovalova MA , Hicks BM , Iacono WG , McGue M . Stability, change, and heritability of borderline personality disorder traits from adolescence to adulthood: a longitudinal twin study. Dev Psychopathol. 2009;21(4):1335‐1353. 19825271 10.1017/S0954579409990186 PMC2789483

[7] Grant BF , Chou SP , Goldstein RB , et al. Prevalence, correlates, disability, and comorbidity of DSM‐IV borderline personality disorder: results from the wave 2 National Epidemiologic Survey on alcohol and related conditions. J Clin Psychiatry. 2008;69(4):533‐545. 18426259 10.4088/jcp.v69n0404 PMC2676679

[8] Zanarini MC , Frankenburg FR , Reich DB , Fitzmaurice GM . Fluidity of the Subsyndromal phenomenology of borderline personality disorder over 16 years of prospective follow‐up. Am J Psychiatry. 2016;173(7):688‐694. 26869248 10.1176/appi.ajp.2015.15081045 PMC4930411

[9] Skoglund C , Tiger A , Ruck C , et al. Familial risk and heritability of diagnosed borderline personality disorder: a register study of the Swedish population. Mol Psychiatry. 2019;26:999‐1008. 31160693 10.1038/s41380-019-0442-0 PMC7910208

[10] Philipsen A , Limberger MF , Lieb K , et al. Attention‐deficit hyperactivity disorder as a potentially aggravating factor in borderline personality disorder. Br J Psychiatry. 2008;192(2):118‐123. 18245028 10.1192/bjp.bp.107.035782

[11] Weibel S , Nicastro R , Prada P , et al. Screening for attention‐deficit/hyperactivity disorder in borderline personality disorder. J Affect Disord. 2018;226:85‐91. 28964997 10.1016/j.jad.2017.09.027

[12] Amad A , Ramoz N , Thomas P , Jardri R , Gorwood P . Genetics of borderline personality disorder: systematic review and proposal of an integrative model. Neurosci Biobehav Rev. 2014;40:6‐19. 24456942 10.1016/j.neubiorev.2014.01.003

[13] Distel MA , Carlier A , Middeldorp CM , Derom CA , Lubke GH , Boomsma DI . Borderline personality traits and adult attention‐deficit hyperactivity disorder symptoms: a genetic analysis of comorbidity. Am J Med Genet B Neuropsychiatr Genet. 2011;156B(7):817‐825. 21812103 10.1002/ajmg.b.31226 PMC3990457

[14] Kuja‐Halkola R , Lind Juto, K. , Skoglund, C. , Rück, C. , Mataix‐Cols, D. , Pérez‐Vigil, A. , Larsson, J. , Hellner, C. , Långström, N. , Petrovic, P. , Lichtenstein, P. , Larsson, H. Do borderline personality disorder and attention‐deficit/hyperactivity disorder co‐aggregate in families? A population‐based study of 2 million swedes. Mol Psychiatry. 2018;26:341‐349. 30323291 10.1038/s41380-018-0248-5 PMC7815504

[15] Storebo OJ , Simonsen E . Is ADHD an early stage in the development of borderline personality disorder? Nord J Psychiatry. 2014;68(5):289‐295. 24117059 10.3109/08039488.2013.841992

[16] Matthies S , Philipsen A . Comorbidity of personality disorders and adult attention deficit hyperactivity disorder (ADHD)‐review of recent findings. Curr Psychiatry Rep. 2016;18(4):33. 26893231 10.1007/s11920-016-0675-4

[17] Fossati A , Novella L , Donati D , Donini M , Maffei C . History of childhood attention deficit/hyperactivity disorder symptoms and borderline personality disorder: a controlled study. Compr Psychiatry. 2002;43(5):369‐377. 12216012 10.1053/comp.2002.34634

[18] Miller CJ , Flory JD , Miller SR , Harty SC , Newcorn JH , Halperin JM . Childhood attention‐deficit/hyperactivity disorder and the emergence of personality disorders in adolescence: a prospective follow‐up study. J Clin Psychiatry. 2008;69(9):1477‐1484. 19193347 10.4088/jcp.v69n0916 PMC2637402

[19] Stepp SD , Burke JD , Hipwell AE , Loeber R . Trajectories of attention deficit hyperactivity disorder and oppositional defiant disorder symptoms as precursors of borderline personality disorder symptoms in adolescent girls. J Abnorm Child Psychol. 2012;40(1):7‐20. 21671009 10.1007/s10802-011-9530-6 PMC3214601

[20] Fossati A , Gratz KL , Borroni S , Maffei C , Somma A , Carlotta D . The relationship between childhood history of ADHD symptoms and DSM‐IV borderline personality disorder features among personality disordered outpatients: the moderating role of gender and the mediating roles of emotion dysregulation and impulsivity. Compr Psychiatry. 2015;56:121‐127. 25446725 10.1016/j.comppsych.2014.09.023

[21] World Health Organization . ICD‐10 Classification of Mental and Behavioural Disorders: Clinical Descriptions and Diagnostic Guidelines. World Health Organization; 1992.

[22] World Health Organization ICD‐10 Classification of Mental and Behavioural Disorders: Diagnostic Criteria for Research. World Health Organization; 1993.

[23] Ronald A , Larsson H , Anckarsater H , Lichtenstein P . Symptoms of autism and ADHD: a Swedish twin study examining their overlap. J Abnorm Psychol. 2014;123(2):440‐451. 24731073 10.1037/a0036088

[24] Gomez R . ADHD and hyperkinetic disorder symptoms in Australian adults: descriptive scores, incidence rates, factor structure, and gender invariance. J Atten Disord. 2016;20(4):325‐334. 23628968 10.1177/1087054713485206

[25] Hinshaw SP , Owens EB , Zalecki C , et al. Prospective follow‐up of girls with attention‐deficit/hyperactivity disorder into early adulthood: continuing impairment includes elevated risk for suicide attempts and self‐injury. J Consult Clin Psychol. 2012;80(6):1041‐1051. 22889337 10.1037/a0029451 PMC3543865

[26] Quinn PD , Pettersson E , Lundstrom S , et al. Childhood attention‐deficit/hyperactivity disorder symptoms and the development of adolescent alcohol problems: a prospective, population‐based study of Swedish twins. Am J Med Genet B Neuropsychiatr Genet. 2016;171(7):958‐970. 26714985 10.1002/ajmg.b.32412 PMC5300044

[27] van Dijk F , Lappenschaar M , Kan C , Verkes RJ , Buitelaar J . Lifespan attention deficit/hyperactivity disorder and borderline personality disorder symptoms in female patients: a latent class approach. Psychiatry Res. 2011;190(2–3):327‐334. 21794926 10.1016/j.psychres.2011.06.023

[28] Anckarsäter H , Lundström S , Kollberg L , et al. The child and adolescent twin study in Sweden (CATSS). Twin Res Hum Genet. 2011;14(6):495‐508. 22506305 10.1375/twin.14.6.495

[29] Zagai U , Lichtenstein P , Pedersen NL , Magnusson PKE . The Swedish twin registry: content and management as a research infrastructure. Twin Res Hum Genet. 2019;22(6):672‐680. 31747977 10.1017/thg.2019.99

[30] Ludvigsson JF , Andersson E , Ekbom A , et al. External review and validation of the Swedish national inpatient register. BMC Public Health. 2011;11:450. 21658213 10.1186/1471-2458-11-450 PMC3142234

[31] Marland C , Lichtenstein P , Degl'Innocenti A , et al. The autism‐tics, ADHD and other comorbidities inventory (A‐TAC): previous and predictive validity. BMC Psychiatry. 2017;17(1):403. 29246205 10.1186/s12888-017-1563-0 PMC5732476

[32] American Psychiatric Association. Diagnostic and Statistical Manual of Mental Disorders IV. In:1994.

[33] Swanson JM , Sergeant JA , Taylor E , Sonuga‐Barke EJ , Jensen PS , Cantwell DP . Attention‐deficit hyperactivity disorder and hyperkinetic disorder. Lancet. 1998;351(9100):429‐433. 9482319

[34] Larson T , Kerekes N , Selinus EN , et al. Reliability of autism‐tics, AD/HD, and other comorbidities (A‐TAC) inventory in a test‐retest design. Psychol Rep. 2014;114(1):93‐103. 24765712 10.2466/03.15.PR0.114k10w1

[35] Ekselius L . Personlighetssyndrom: kliniska riktlinjer för utredning och behandling. Första upplagan ed. Svenska psykiatriska föreningen; 2017.

[36] Kouppis E , Ekselius L . Validity of the personality disorder diagnosis in the Swedish National Patient Register. Acta Psychiatr Scand. 2020;141(5):432‐438. 32092153 10.1111/acps.13166

[37] Wettermark B , Hammar N , Fored CM , et al. The new Swedish prescribed drug register‐‐opportunities for pharmacoepidemiological research and experience from the first six months. Pharmacoepidemiol Drug Saf. 2007;16(7):726‐735. 16897791 10.1002/pds.1294

[38] Kessler RC , Adler L , Ames M , et al. The World Health Organization adult ADHD self‐report scale (ASRS): a short screening scale for use in the general population. Psychol Med. 2005;35(2):245‐256. 15841682 10.1017/s0033291704002892

[39] D'Onofrio BM , Sjolander A , Lahey BB , Lichtenstein P , Oberg AS . Accounting for confounding in observational studies. Annu Rev Clin Psychol. 2020;16:25‐48. 32384000 10.1146/annurev-clinpsy-032816-045030

[40] Therneau T. A Package for Survival Analysis in R. R package version 3.2–10. 2021.

[41] Zetterqvist J , Sjölander A . Doubly robust estimation with the R package drgee. In: Epidemiologic Methods. De Gruyter; 2015:69‐86.

[42] Ditrich I , Philipsen A , Matthies S . Borderline personality disorder (BPD) and attention deficit hyperactivity disorder (ADHD) revisited ‐ a review‐update on common grounds and subtle distinctions. Borderline Personal Disord Emot Dysregul. 2021;8(1):22. 34229766 10.1186/s40479-021-00162-w PMC8261991

[43] Sebastian A , Jung P , Krause‐Utz A , Lieb K , Schmahl C , Tüscher O . Frontal dysfunctions of impulse control ‐ a systematic review in borderline personality disorder and attention‐deficit/hyperactivity disorder. Front Hum Neurosci. 2014;8:698. 25232313 10.3389/fnhum.2014.00698 PMC4153044

[44] Jacobson LA , Crocetti D , Dirlikov B , et al. Anomalous brain development is evident in preschoolers with attention‐deficit/hyperactivity disorder. J Int Neuropsychol Soc. 2018;24(6):531‐539. 29576028 10.1017/S1355617718000103 PMC6035105

[45] Chronis‐Tuscano A , Molina BS , Pelham WE , et al. Very early predictors of adolescent depression and suicide attempts in children with attention‐deficit/hyperactivity disorder. Arch Gen Psychiatry. 2010;67(10):1044‐1051. 20921120 10.1001/archgenpsychiatry.2010.127 PMC3382065

[46] Zetterqvist M , Lundh LG , Dahlstrom O , Svedin CG . Prevalence and function of non‐suicidal self‐injury (NSSI) in a community sample of adolescents, using suggested DSM‐5 criteria for a potential NSSI disorder. J Abnorm Child Psychol. 2013;41(5):759‐773. 23344701 10.1007/s10802-013-9712-5

[47] Kapur N , Cooper J , O'Connor RC , Hawton K . Non‐suicidal self‐injury v. attempted suicide: new diagnosis or false dichotomy? Br J Psychiatry. 2013;202(5):326‐328. 23637107 10.1192/bjp.bp.112.116111

[48] Yip SW , Potenza MN . Application of research domain criteria to childhood and adolescent impulsive and addictive disorders: implications for treatment. Clin Psychol Rev. 2018;64:41‐56. 27876165 10.1016/j.cpr.2016.11.003 PMC5423866

[49] Bohn MJ , Babor TF , Kranzler HR . The alcohol use disorders identification test (AUDIT): validation of a screening instrument for use in medical settings. J Stud Alcohol. 1995;56(4):423‐432. 7674678 10.15288/jsa.1995.56.423

[50] Carver CS , Johnson SL . Impulsive reactivity to emotion and vulnerability to psychopathology. Am Psychol. 2018;73(9):1067‐1078. 30525782 10.1037/amp0000387 PMC6309622

[51] Pettersson E , Anckarsäter H , Gillberg C , Lichtenstein P . Different neurodevelopmental symptoms have a common genetic etiology. J Child Psychol Psychiatry. 2013;54(12):1356‐1365. 24127638 10.1111/jcpp.12113

