# Supplementary material for: Childhood symptoms of attention‐deficit/hyperactivity disorder and borderline personality disorder
Source: Acta Psychiatr Scand. 2022 Jul 23;146(4):370–80. doi: 10.1111/acps.13476 (PMC9796766; doi:10.1111/acps.13476)
Supplement: Supplementary file 1 — Appendix S1 Supporting Information [file ACPS-146-370-s001.docx]

**Childhood symptoms of Attention-Deficit/Hyperactivity Disorder and Borderline Personality Disorder**

**Supplementary Online material**

Annika Tiger, M.D.^1^, Anna Ohlis, M.D., Ph.D.^1,2^, Johan Bjureberg, Ph.D.^1,3^, Sebastian Lundström, Ph.D.^4^, Paul Lichtenstein, Ph.D.^5^, Henrik Larsson, Ph.D.^5,6^, Clara Hellner, M.D. Ph.D.^1^, Ralf Kuja-Halkola, Ph.D.^5^, Nitya Jayaram-Lindström, Ph.D.^1^

**These authors contributed equally:** Ralf Kuja-Halkola and Nitya Jayaram-Lindström

**Author affiliations:**

^1^ Centre for Psychiatry Research, Department of Clinical Neuroscience, Karolinska Institutet & Stockholm Health Care Services, Region Stockholm, Stockholm, Sweden

^2^ Centre for Epidemiology and Community Medicine & Stockholm Health Care Services, Region Stockholm, Stockholm, Sweden

^3^ Department of Psychology, Stanford University, Stanford, California, USA.

^4^ Department of Neuroscience and Physiology, Sahlgrenska Academy, Göteborg, Sweden

^5^ Department of Medical Epidemiology and Biostatistics, Karolinska Institutet, Stockholm, Sweden.

^6^ Department of Medical Sciences, Örebro University, Örebro, Sweden

**Corresponding author:**

Annika Tiger, Centre for Psychiatry Research, Department of Clinical Neuroscience, Karolinska Institutet, Norra Stationsgatan 69, SE-113 64 Stockholm, Sweden. E-mail: annika.tiger@ki.se

**FigureS1**. Flow chart of study cohort selection

**FigureS2.** Plot of eigenvalues/total variances by principal components 1 to 8 (of 18) in principal component analysis.

Dotted, grey line at eigenvalue/total variance 1 indicating that those above have items correlating into the indicated number of components better than by chance. The plot hence indicates that the items of A-TAC correlate in a 3-factor structure.

The number of subjects in the principal component analysis was 33 927, i.e. those included in the CATSS 9/12 cohort (N=33 976), excluding those with incomplete age 9/12 ADHD symptom variable data in the A-TAC (N=49).

**TableS1.** Loadings of ADHD Symptom Items in principal component analysis.

The varimax rotation grouped the loadings of ADHD-items into subdimensions inattention, hyperactivity and impulsivity. Sex and age-groups are combined. Loadings > 0.4 in bold.

| **Component/Item** | **1** | **2** | **3** |
| --- | --- | --- | --- |
| *Inattention* |  |  |  |
| Does s/he often fail to pay close attention to details or make careless mistakes in schoolwork, or other activities? | **0.68** | 0.18 | 0.15 |
| Does s/he often have difficulty sustaining attention in tasks or play activities? | **0.74** | 0.35 | 0.12 |
| Does s/he often seem not to listen when spoken to directly? | **0.66** | 0.27 | 0.25 |
| Does s/he have difficulty following instructions and to finish tasks? | **0.75** | 0.29 | 0.06 |
| Does s/he often have difficulty organizing tasks and activities? | **0.75** | 0.27 | 0.08 |
| Does s/he often avoid tasks that require sustained mental effort (such as homework)? | **0.70** | 0.25 | 0.06 |
| Does s/he often lose things? | **0.65** | 0.01 | 0.28 |
| Is s/he often easily distracted or disturbed? | **0.70** | 0.28 | 0.21 |
| Is s/he often forgetful in daily activities? | **0.69** | 0.01 | 0.23 |
| *Hyperactivity* |  |  |  |
| Does s/he have difficulties holding his/her hands and feet still or can s/he not stay seated? | 0.27 | **0.67** | 0.25 |
| Does s/he get up and move about in school or in other situations when s/he is supposed to remain seated? | 0.34 | **0.63** | 0.20 |
| Does s/he often run around and climbs more than his/hers peers? | 0.16 | **0.74** | 0.17 |
| Does s/he have difficulty playing calmly and quietly? | 0.20 | **0.70** | 0.20 |
| Is s/he often “on the go” or does s/he often act as if driven by a motor? | 0.18 | **0.77** | 0.26 |
| *Impulsivity* |  |  |  |
| Does s/he talk excessively? | 0.10 | 0.19 | **0.72** |
| Does s/he often blurt out answers before the question has been completed? | 0.16 | 0.20 | **0.76** |
| Does s/he have difficulty awaiting turns? | 0.28 | 0.33 | **0.68** |
| Does s/he often interrupt or intrude on others? | 0.26 | 0.27 | **0.73** |

**TableS2.** Chart of questionnaires used, all self-rating questionnaires except for the Adult Behavior Check List that is a parent-rating questionnaire

| Name of Questionnaire | Item used | Questionnaire response options and corresponding variable *value* | Variable name and definition |
| --- | --- | --- | --- |
| Life History of Aggression questionnaire (LHA) | 6) “How many times in life so far, would you say that you have deliberately attempted to injure yourself physically when you were angry or despondent” | no event =0  one event=1,  2-3 events=2,  4-9 events=3  10+ events=4 | Self-harm  “Ever” having self-harmed was defined as having a “positive” response, i.e. a response value ≥ 0 to any of the four self-harm questionnaire items even if any of the other self-harm items had missing responses and “never” if responses were only “negative”, = 0. If all items lacked responses the variable was indicated as missing |
|  | 7) “How many times in life so far, would you say that you deliberately attempted to kill yourself when you were angry or despondent” |  |  |
| Brief Obsessive Compulsive Scale | 15) “I do things that injure my body”. | Never=0  Current=1  Past=2 |  |
| Adult Behavior Check List (parent-rating questionnaire) | 18) “Deliberately harms self or attempts suicide” | Not true=0  Somewhat or sometimes true=1,  very true or often true=2 |  |
| Alcohol Use Disorders Identification Test (AUDIT) | 1) “How often do you have a drink containing alcohol?” | Never=0,  Monthly or less=1,  2-4 times a month=2,  2-3 times a week=3,  4 or more times a week=4 | Alcohol use  Extent of alcohol use was defined as the summed up score, ranging between 0 and 40, for all AUDIT items |
|  | 2) How many drinks containing alcohol do you have on a typical day when you are drinking? | 1-2=0,  3-4=1,  5-6=2,  7-9=3,  10 or more=4 |  |
|  | 3) How often do you have six or more drinks on one occasion? | Never=0,  less than monthly=1,  monthly=2,  weekly=3,  daily or almost daily=4 |  |
|  | 4) How often during the last year have you found that you were not able to stop drinking once you had started? |  |  |
|  | 5) How often during the last year have you failed to do what was normally expected from you because of drinking? |  |  |
|  | 6) How often during the last year have you needed a first drink in the moming to get yourself going after a heavy drinking session? |  |  |
|  | 7) How often during the last year have you had a feeling of guilt or remorse after drinking? |  |  |
|  | 8) How often during the last year have you been unable to remember what happened the night before because you  had been drinking? |  |  |
|  | 9) Have you or someone else been injured as a result of your drinking? | No=0, yes, but not in the last year=2, yes, during the last year=4 |  |
|  | 10) Has a relative or friend, or a doctor or other health worker been concerned about your drinking or suggested  you cut down? |  |  |
| DUDIT (Drug Use Disorders Identification Test) | 1) How often do you use drugs other than alcohol? | Never=0, once a month or less often=1, 2-4 times a month=2, 2-3 times a week=3, 4 times a week or more often=4 | Drug use  Extent of drug use was defined as the summed up score, ranging between 0 and 44, for all DUDIT items |
|  | 2) Do you use more than one type of drug on the same occasion? |  |  |
|  | 3) How many times do you take drugs on a typical day when you use drugs? | 0 times=0, 1-2 times=1, 3-4 times=2, 5-6 times=3, 7 or more times=4 |  |
|  | 4) How often are you influenced heavily by drugs? | Never=0, less often than once a month=1, every month=2, every week=3, daily or almost every day=4 |  |
|  | 5) Over the past year, have you felt that your longing for drugs was so strong that you could not resist it? |  |  |
|  | 6) Has it happened, over the past year, that you have not been able to stop taking drugs once you started? |  |  |
|  | 7) How often over the past year have you taken drugs and then neglected to do something you should have done? |  |  |
|  | 8) How often over the past year have you needed to take a drug the morning after heavy drug use the day before? |  |  |
|  | 9) How often over the past year have you had guilt feelings or a bad conscience because you used drugs? |  |  |
|  | 10) Have you or anyone else been hurt (mentally or physically) because you used drugs? | No=0, yes, but not in the last year=2, yes, during the last year=4 |  |
|  | 11) Has a relative or a friend, a doctor or a nurse, or anyone else, been worried about your drug use or said to you that you should stop using drugs? |  |  |
| ASRS (Adult ADHD Self-Report Scale) | 1) How often do you have trouble wrapping up the fine details of a project, once the challenging parts have been done? | 1=never  2=rarely  3=sometimes  4=often  5=very often | Age 18 ADHD-symptomathology;  Total ADHD score = sum of ratings for questions 1-18  Inattention=sum of ratings for questions 1, 2, 3, 4, 7, 8, 9, 10 and 11  Hyperactivity = sum of ratings for questions 5, 6, 12, 13 and 14  Impulsivity = sum of ratings for questions 15, 16, 17 and 18 |
|  | 2) How often do you have difficulty getting things in order when you have to do a task that requires organization? |  |  |
|  | 3) How often do you have problems remembering appointments or obligations ? |  |  |
|  | 4) When you have a task that requires a lot of thought, how often do you avoid or delay getting started? |  |  |
|  | 5) How often do you fidget or squirm with your hands or your feet when you have to sit down for a long time? |  |  |
|  | 6) How often do you feel overly active and compelled to do things, like you were driven by a motor? |  |  |
|  | 7) How often do you make careless mistakes when you have to work on a boring or difficult project ? |  |  |
|  | 8) How often do you have difficulty keeping your attention when you are doing boring or repetitive work? |  |  |
|  | 9) How often do you have difficulty concentrating on what people say to you, even when they are speaking to you directly ? |  |  |
|  | 10) How often do you misplace or have difficulty finding things at home or at work? |  |  |
|  | 11) How often are you distracted by activity or noise around you? |  |  |
|  | 12) How often do you leave your seat in meetings or other situations in which you are expected to remain seated ? |  |  |
|  | 13) How often do you feel restless or fidgety? |  |  |
|  | 14) How often do you have difficulty unwinding and relaxing when you have time to yourself ? |  |  |
|  | 15) How often do you find yourself talking too much when you are in a social situation ? |  |  |
|  | 16) When you’re in a conversation, how often do you find yourself finishing the sentences of the people that you are talking to, before they can finish them themselves ? |  |  |
|  | 17) How often do you have difficulty waiting your turn in situations when turn-taking is required? |  |  |
|  | 18) How often do you interrupt others when they are busy? |  |  |

**TableS3.** Distribution of positive responses, i.e. reporting having self-harmed in the questionnaires used for the variable self-harm.

|  | Total (N=8995) | female (n=5209) | male (n=3786) |
| --- | --- | --- | --- |
| Self-harm, N (%) | 2622 (29.1%) | 1827 (35.1%) | 795 (21.0%) |
| Brief Obsessive Compulsive Scale, n (%) *missingness* | 1047 (12.0%) *279* | 853 (16.8%)  *128* | 194 (5.3%)  *151* |
| Life History of Agression 6 & 7, n (%) *missingness* | 2496 (27.8%) *30* | 1741 (33.6%)  *22* | 755 (20.0%)  *8* |
| Life Histpry of aggression 6, n (%) *missingness* | 2437 (27.2%) *25* | 1710 (32.9%)  *16* | 727 (19.2%)  *9* |
| Life History of aggression 7, n (%) *missingness* | 530 (5.9%)  *28* | 394 (7.6%)  *18* | 136 (3.6%)  *10* |
| Adult Behavior Check List, n (%) *missingness* | 93 (1.5%)  *2714* | 82 (2.3%)  *1715* | 11 (0.4%)  *999* |

**TableS4.** Medications for ADHD that were considered.

Having been expedited any three filled prescriptions before childhood assessment defined “medication in childhood”, any time between childhood assessment and BPD defined “medication before BPD” and two years before secondary outcomes defined “medication before secondary outcomes”.

| Name of medication | ATC-code |
| --- | --- |
| Amphetamine | N06BA01 |
| Dexamphetamine | N06BA02 |
| Metamphetamine | N06BA03 |
| Metylphenidate | [N06BA04](https://www.fass.se/LIF/atcregister?atcCode=N06BA04) |
| Modafinil | N06BA07 |
| Atomoxetine | N06BA09 |
| Lisdexamphetamine | N06BA12 |

**FigureS3.** Associations between symptoms of ADHD (**non-standardized**) with adjusted estimates.

Separate models on left side, combined models on right side.

**a)** Hazard ratios with 95% CI with BPD as outcome for total population


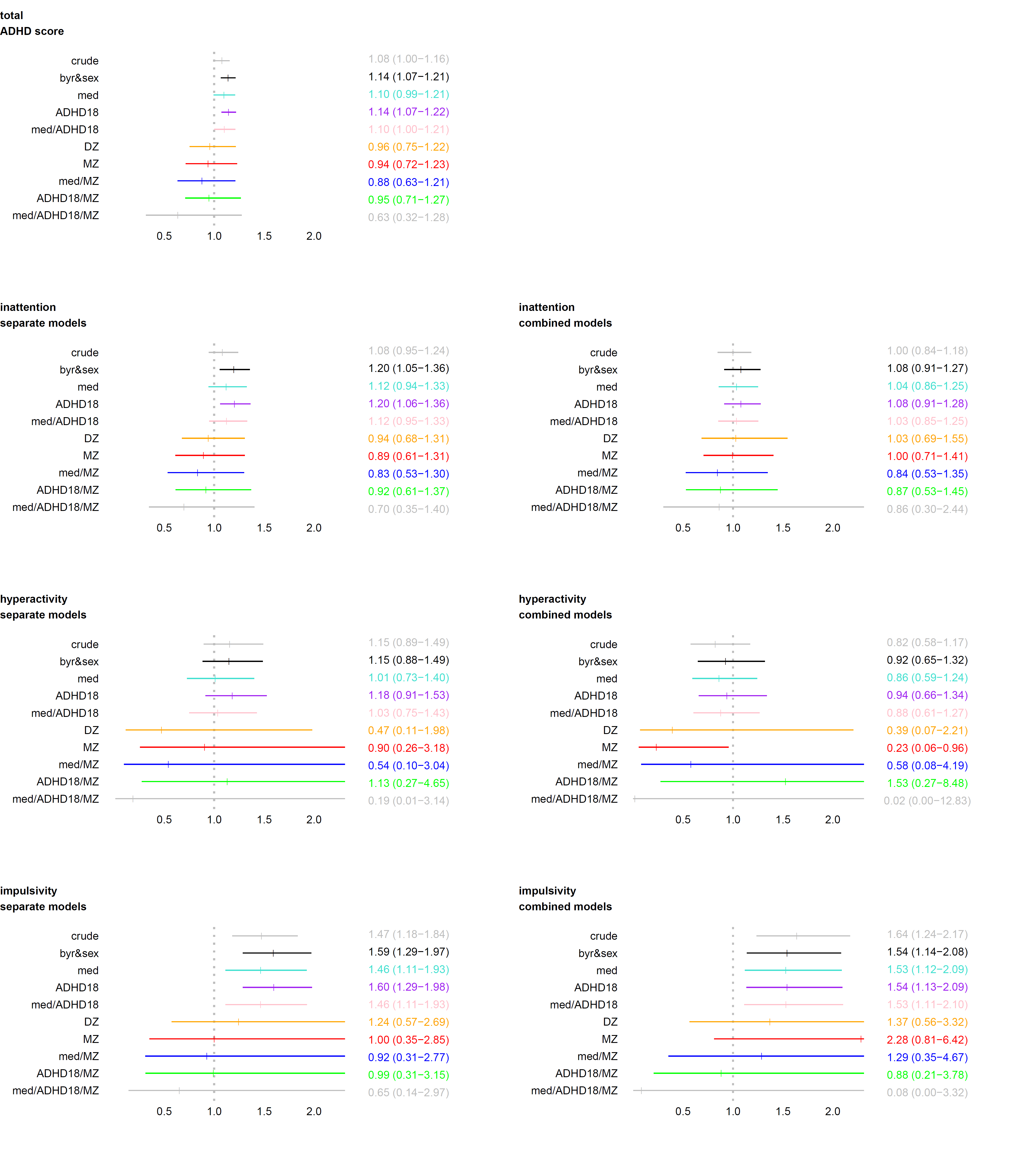


Extreme CI-values have been cut in figure.

Model adjustment abbreviations explained:

**crude**= unadjusted model, **byr&sex** = adjustment for birth-year and sex, **med** = adjustment for birth-year, sex and medication before BPD, **ADHD18** = adjustment for birth-year, sex and symptoms of ADHD at age 18, **DZ** = adjustment for birth-year, sex and shared stable confounders within twin pairs, for dizygotic twins, **MZ** = adjustment for birth-year, sex and shared stable confounders within twin pairs, for monozygotic twins.

Adjustment for medication in childhood was omitted in BPD analyses as individuals medicating in childhood (i.e., before ADHD assessment at age 9/12) were few and no meaningful estimate could be obtained.

**b)** Hazard ratios with 95% CI with BPD as outcome for females only


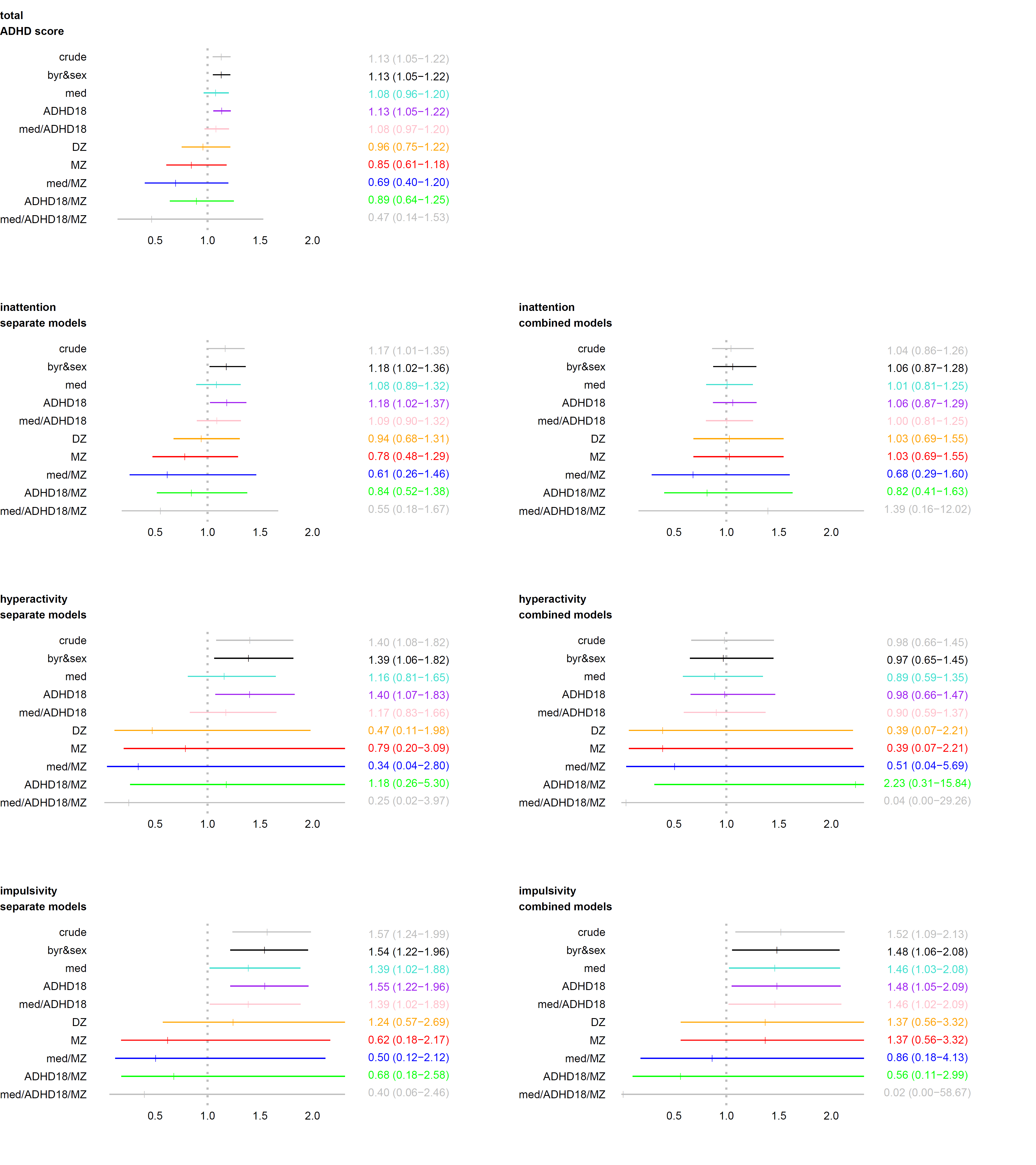


Extreme CI-values have been cut in figure.

Model adjustment abbreviations explained:

**crude**= unadjusted model, **byr&sex** = adjustment for birth-year and sex, **med** = adjustment for birth-year, sex and medication before BPD, **ADHD18** = adjustment for birth-year, sex and symptoms of ADHD at age 18, **DZ** = adjustment for birth-year, sex and shared stable confounders within twin pairs, for dizygotic twins, **MZ** = adjustment for birth-year, sex and shared stable confounders within twin pairs, for monozygotic twins.

Adjustment for medication in childhood was omitted in BPD analyses of female as individuals medicating in childhood (i.e., before ADHD assessment at age 9/12) were few and no meaningful estimate could be obtained.

**c)** Odds ratios with 95% CI with self-harm as outcome for total population


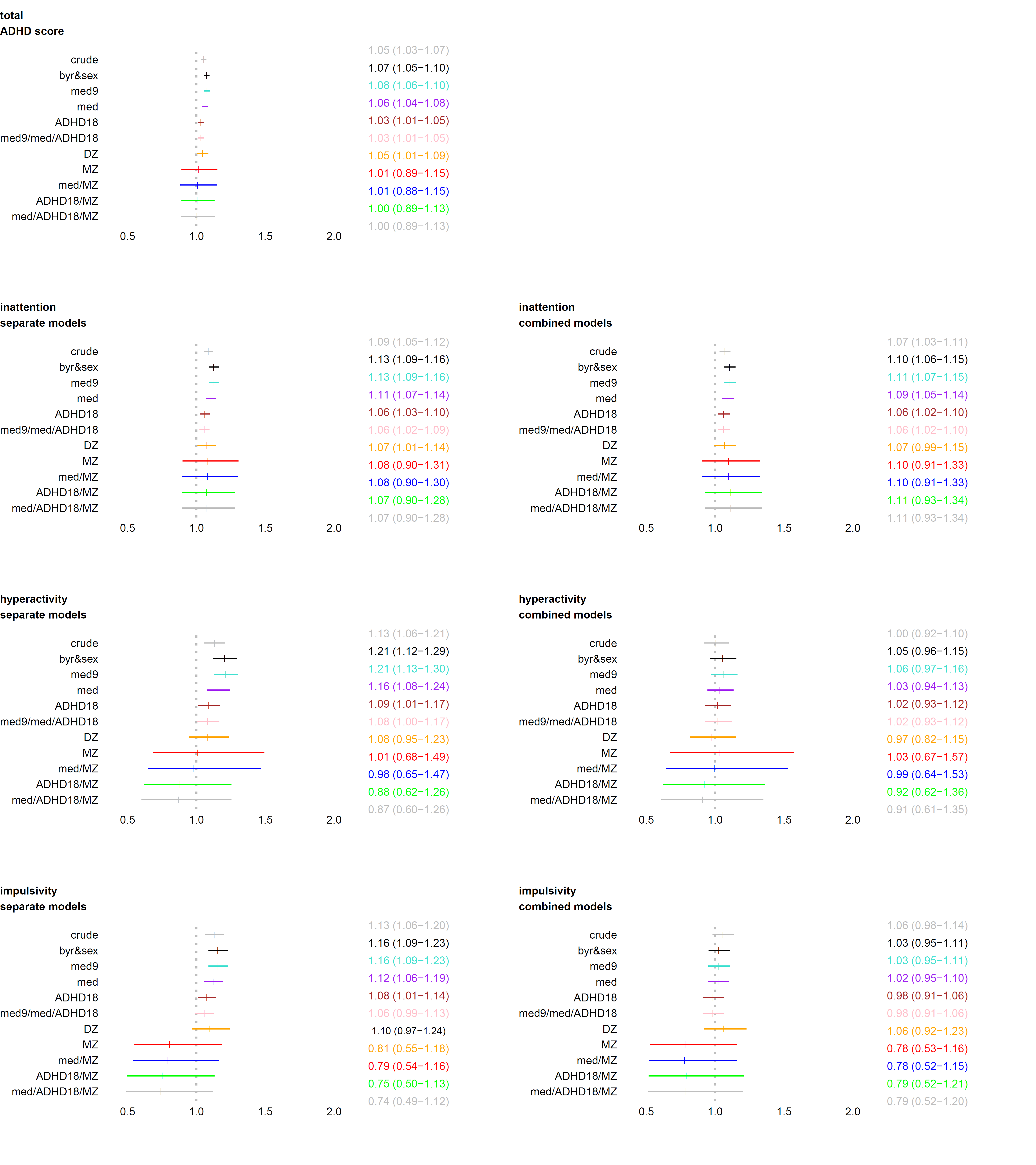


Extreme CI-values have been cut in figure.

Model adjustment abbreviations explained:

**crude**= unadjusted model, **byr&sex** = adjustment for birth-year and sex, **med9** = adjustment for birth-year, sex and medication in childhood, **med** = adjustment for birth-year, sex and medication before age 18, **ADHD18** = adjustment for birth-year, sex and symptoms of ADHD at age 18, **DZ** = adjustment for birth-year, sex and shared stable confounders within twin pairs, for dizygotic twins, **MZ** = adjustment for birth-year, sex and shared stable cnfounders within twin pairs, for monozygotic twins.

**d)** Associations (regression coefficients) with 95% CI with AUDIT as outcome for total population


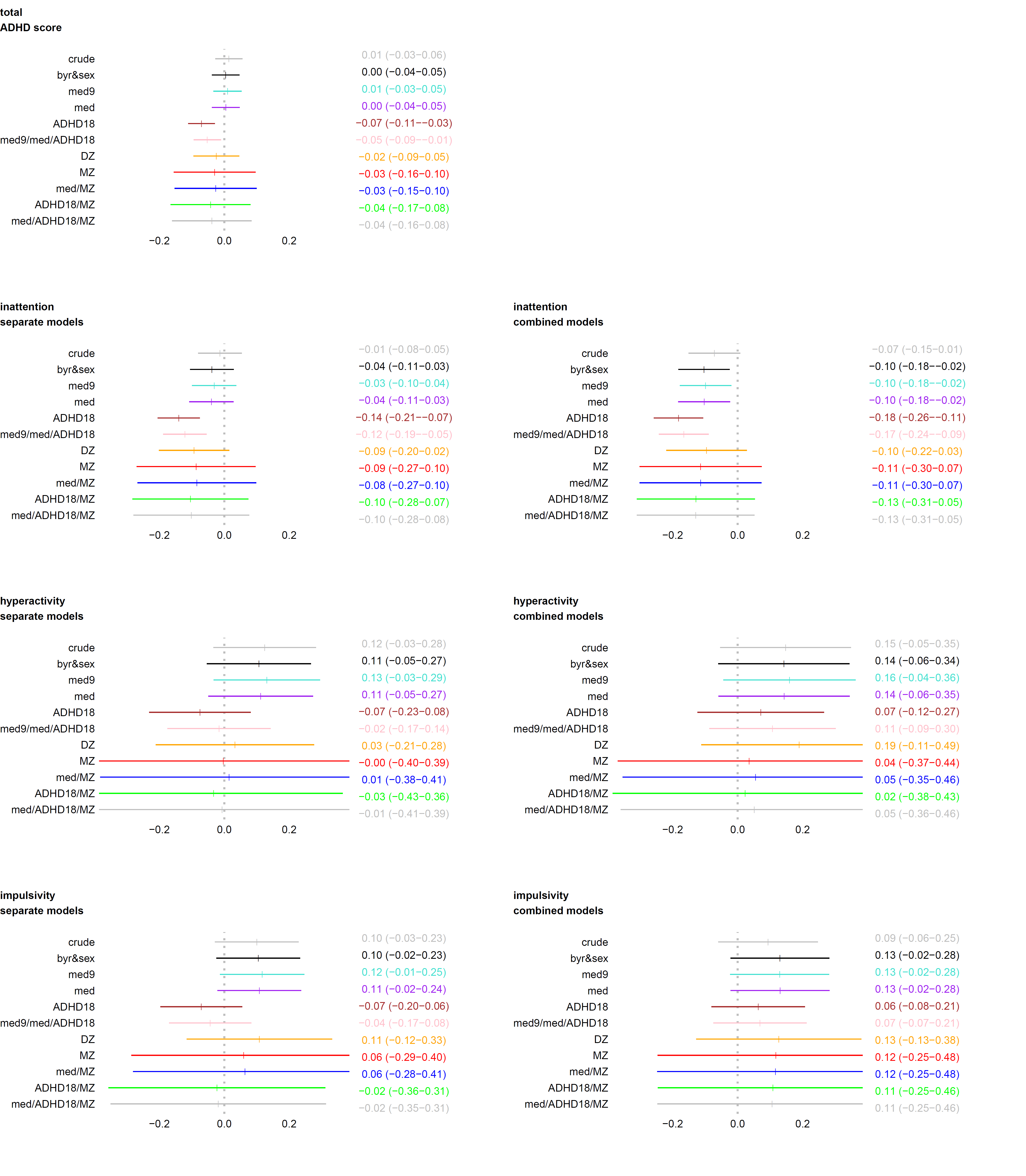


Extreme CI-values have been cut in figure.

Model adjustment abbreviations explained:

**crude**= unadjusted model, **byr&sex** = adjustment for birth-year and sex, **med9** = adjustment for birth-year, sex and medication in childhood, **med** = adjustment for birth-year, sex and medication before age 18, **ADHD18** = adjustment for birth-year, sex and symptoms of ADHD at age 18, **DZ** = adjustment for birth-year, sex and shared stable confounders within twin pairs, for dizygotic twins, **MZ** = adjustment for birth-year, sex and shared stable confounders within twin pairs, for monozygotic twins.

**e)** Associations (regression coefficients) with 95% CI with DUDIT as outcome for total population


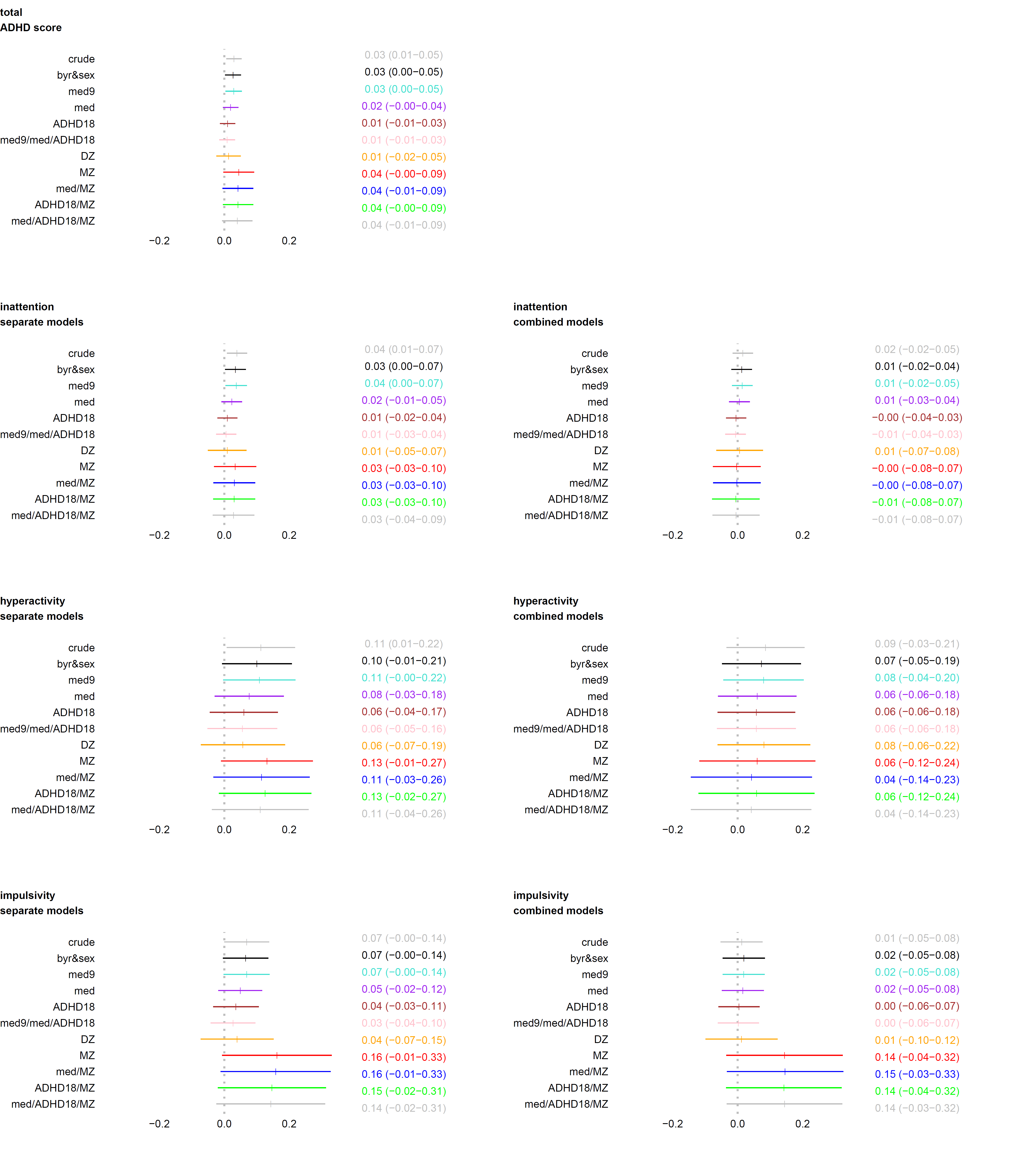


Extreme CI-values have been cut in figure.

Model adjustment abbreviations explained:

**crude**= unadjusted model, **byr&sex** = adjustment for birth-year and sex, **med9** = adjustment for birth-year, sex and medication in childhood, **med** = adjustment for birth-year, sex and medication before age 18, **ADHD18** = adjustment for birth-year, sex and symptoms of ADHD at age 18, **DZ** = adjustment for birth-year, sex and shared stable confounders within twin pairs, for dizygotic twins, **MZ** = adjustment for birth-year, sex and shared stable confounders within twin pairs, for monozygotic twins.
